# Ethyl 4-anilino-3-nitro­benzoate

**DOI:** 10.1107/S1600536812019903

**Published:** 2012-05-12

**Authors:** Yeong Keng Yoon, Elumalai Manogaran, Mohamed Ashraf Ali, Suhana Arshad, Ibrahim Abdul Razak

**Affiliations:** a Institute for Research in Molecular Medicine, Universiti Sains Malaysia, Minden 11800, Penang, Malaysia; b Faculty of Pharmaceutical Sciences, UCSI University, Kuala Lumpur, Cheras 56000, Malaysia; c School of Physics, Universiti Sains Malaysia, 11800 USM, Penang, Malaysia

## Abstract

In the title compound, C_15_H_14_N_2_O_4_, the dihedral angle between the benzene and phenyl rings is 73.20 (6)°. An intra­molecular N—H⋯O hydrogen bond forms an *S*(6) ring motif. In the crystal, mol­ecules are linked by N—H⋯O and C—H⋯O hydrogen bonds into a layer parallel to the *bc* plane.

## Related literature

For applications of nitro­phenyl­ene­amines, see: Stephane (2006 ▶); Glebowska *et al.* (2009 ▶); Remusat *et al.* (2004 ▶). For related structures, see: Mohdaidin *et al.* (2008 ▶); Zhang *et al.* (2009 ▶). For hydrogen-bond motifs, see: Bernstein *et al.* (1995 ▶). For the stability of the temperature controller used for the data collection, see: Cosier & Glazer (1986 ▶).
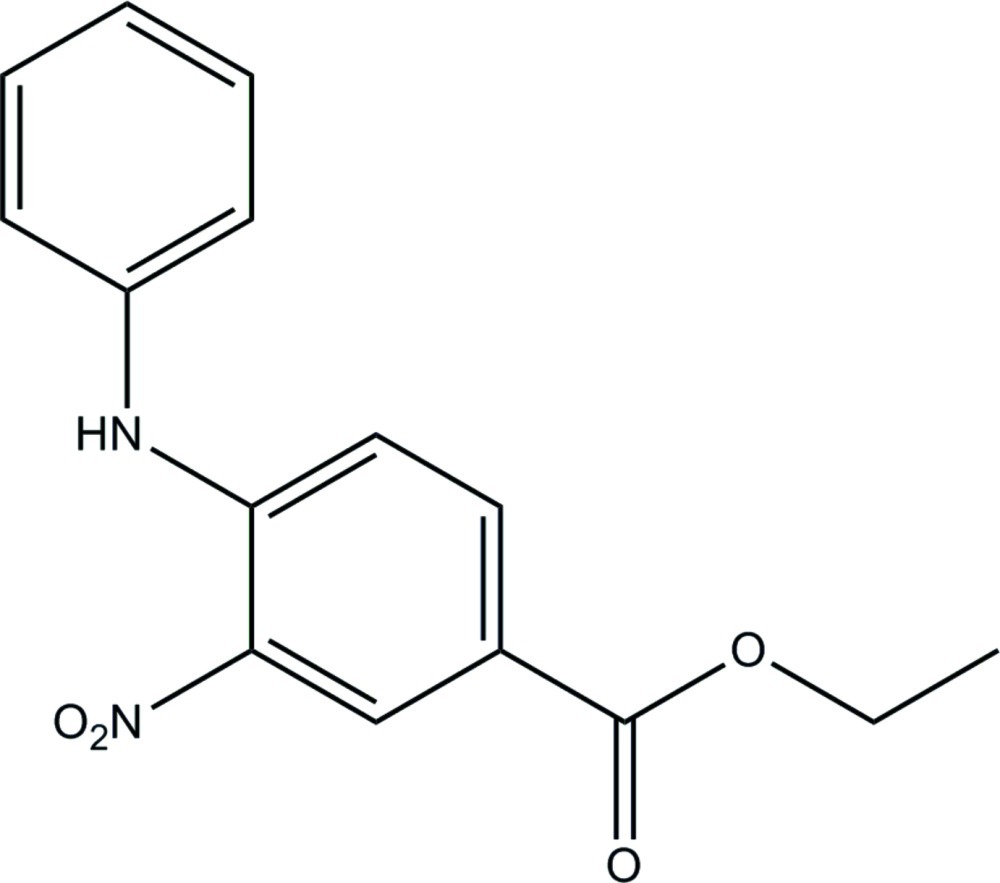


## Experimental

### 

#### Crystal data

C_15_H_14_N_2_O_4_
*M* *_r_* = 286.28
Monoclinic, 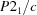

*a* = 10.6464 (2) Å
*b* = 9.9178 (2) Å
*c* = 14.7885 (2) Å
β = 120.244 (1)°
*V* = 1348.96 (4) Å^3^
*Z* = 4
Mo *K*α radiation
μ = 0.10 mm^−1^
*T* = 100 K
0.35 × 0.20 × 0.16 mm

#### Data collection

Bruker SMART APEXII CCD area-detector diffractometer
Absorption correction: multi-scan (*SADABS*; Bruker, 2009 ▶) *T*_min_ = 0.965, *T*_max_ = 0.984
17988 measured reflections
4639 independent reflections
3276 reflections with *I* > 2σ(*I*)
*R*_int_ = 0.040

#### Refinement

*R*[*F* ^2^ > 2σ(*F* ^2^)] = 0.047
*wR*(*F* ^2^) = 0.124
*S* = 1.04
4639 reflections
195 parameters
H atoms treated by a mixture of independent and constrained refinement
Δρ_max_ = 0.39 e Å^−3^
Δρ_min_ = −0.26 e Å^−3^

### 

Data collection: *APEX2* (Bruker, 2009 ▶); cell refinement: *SAINT* (Bruker, 2009 ▶); data reduction: *SAINT*; program(s) used to solve structure: *SHELXTL* (Sheldrick, 2008 ▶); program(s) used to refine structure: *SHELXTL*; molecular graphics: *SHELXTL*; software used to prepare material for publication: *SHELXTL* and *PLATON* (Spek, 2009 ▶).

## Supplementary Material

Crystal structure: contains datablock(s) global, I. DOI: 10.1107/S1600536812019903/is5128sup1.cif

Structure factors: contains datablock(s) I. DOI: 10.1107/S1600536812019903/is5128Isup2.hkl

Supplementary material file. DOI: 10.1107/S1600536812019903/is5128Isup3.cml

Additional supplementary materials:  crystallographic information; 3D view; checkCIF report

## Figures and Tables

**Table 1:** Hydrogen-bond geometry (Å, °)

*D*—H⋯*A*	*D*—H	H⋯*A*	*D*⋯*A*	*D*—H⋯*A*
N1—H1*N*1⋯O4	0.859 (18)	2.00 (2)	2.6375 (17)	130.6 (17)
N1—H1*N*1⋯O2^i^	0.858 (17)	2.288 (16)	2.9411 (14)	133.0 (14)
C15—H15*A*⋯O2^ii^	0.93	2.45	3.342 (2)	160

Symmetry codes: (i) 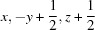; (ii) 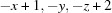.

**Fig. 1.**
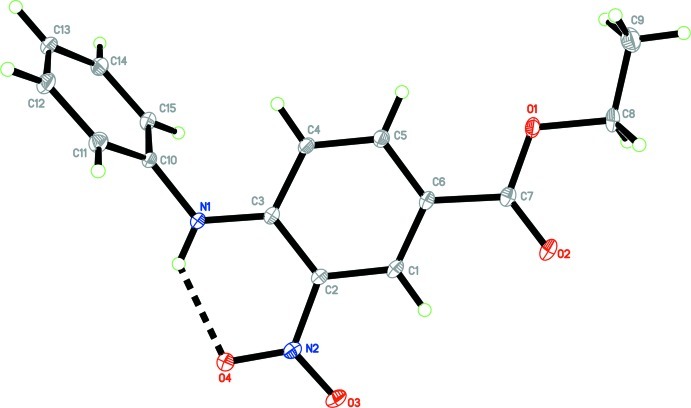
The molecular structure of the title compound, showing 30% probability displacement ellipsoids and the atom-numbering scheme. The intramolecular N—H···O hydrogen bond is shown by a dashed line.

**Fig. 2.**
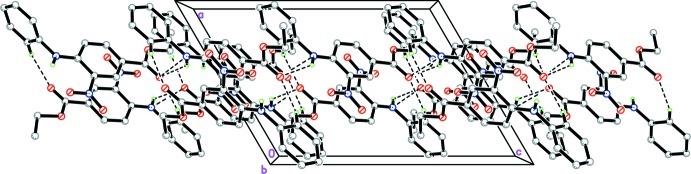
A crystal packing diagram of the title compound, viewed along the b axis. The H atoms not involved in the intermolecular interactions (dashed lines) have been omitted for clarity.

## supplementary crystallographic information

### Comment

In chemistry, nitrophenyleneamines are important building blocks for many pharmaceutical compounds. Phenylenediamine themselves are also used as composition in making dyes (Stephane, 2006), metallomesogens (Glebowska *et al.*, 2009) as well as ligand precursors. Condensation of substituted ο-phenylenediamine with various diketones is then used in the preparation of a variety of pharmaceuticals (Remusat *et al.*, 2004).

In the molecular structure (Fig. 1), an intramolecular N1—H1N1···O4 hydrogen bond generates an *S*(6) ring motif (Bernstein *et al.*, 1995). The C1–C6 benzene ring [maximum deviation of 0.007 (1) Å at atoms C5 and C6] and C10–C15 phenyl ring [maximum deviation of 0.005 (2) Å at atom C12] make a dihedral angle of 73.20 (6)° with each other. Bond lengths and angles are within normal ranges and are comparable to related structures (Mohd. Maidin *et al.*, 2008; Zhang *et al.*, 2009).

The crystal packing is shown in Fig. 2. The intermolecular N1—H1N1···O2 and C15—H15A···O2 (Table 1) hydrogen bonds linked the molecules into a two-dimensional network parallel to the *bc* plane.

### Experimental

Ethyl-4-fluro-3-nitro benzoate (1 g) in dichloromethane (20 ml) was added into the solution of aniline (0.51 g) and *N*,*N*-diisopropylethylamine (0.72 g) in dichloromethane (20 ml). The reaction mixture was stirred overnight at room temperature. After completion of the reaction, evidenced by TLC analysis. The reaction mixture was washed with water (10 ml × 2) and 10% Na_2_CO_3_ (10 ml × 2). The dichloromethane layer was collected and dried over Na_2_SO_4_ and evaporated in vacuo to yield the product. The product was recrystallised from ethyl acetate.

### Refinement

N-bound H atom was located in a difference Fourier map and refined freely [N—H = 0.858 (17) Å]. Other H atoms were positioned geometrically (C—H = 0.93–0.97 Å) and refined using a riding model, with *U*_iso_(H) = 1.2 or 1.5*U*_eq_(C). A rotating group model was applied to the methyl group.

### Crystal data

**Table:**

C_15_H_14_N_2_O_4_	*F*(000) = 600
*M**_r_* = 286.28	*D*_x_ = 1.410 Mg m^−^^3^
Monoclinic, *P*2_1_/*c*	Mo *K*α radiation, λ = 0.71073 Å
Hall symbol: -P 2ybc	Cell parameters from 3629 reflections
*a* = 10.6464 (2) Å	θ = 2.9–31.4°
*b* = 9.9178 (2) Å	µ = 0.10 mm^−^^1^
*c* = 14.7885 (2) Å	*T* = 100 K
β = 120.244 (1)°	Block, yellow
*V* = 1348.96 (4) Å^3^	0.35 × 0.20 × 0.16 mm
*Z* = 4	

### Data collection

**Table:**

Bruker SMART APEXII CCD area-detector diffractometer	4639 independent reflections
Radiation source: fine-focus sealed tube	3276 reflections with *I* > 2σ(*I*)
Graphite monochromator	*R*_int_ = 0.040
φ and ω scans	θ_max_ = 31.9°, θ_min_ = 2.2°
Absorption correction: multi-scan (*SADABS*; Bruker, 2009)	*h* = −15→15
*T*_min_ = 0.965, *T*_max_ = 0.984	*k* = −14→14
17988 measured reflections	*l* = −21→21

### Refinement

**Table:**

Refinement on *F*^2^	Primary atom site location: structure-invariant direct methods
Least-squares matrix: full	Secondary atom site location: difference Fourier map
*R*[*F*^2^ > 2σ(*F*^2^)] = 0.047	Hydrogen site location: inferred from neighbouring sites
*wR*(*F*^2^) = 0.124	H atoms treated by a mixture of independent and constrained refinement
*S* = 1.04	*w* = 1/[σ^2^(*F*_o_^2^) + (0.0561*P*)^2^ + 0.2709*P*] where *P* = (*F*_o_^2^ + 2*F*_c_^2^)/3
4639 reflections	(Δ/σ)_max_ = 0.001
195 parameters	Δρ_max_ = 0.39 e Å^−^^3^
0 restraints	Δρ_min_ = −0.26 e Å^−^^3^

### Special details

**Table:**

Experimental. The crystal was placed in the cold stream of an Oxford Cryosystems Cobra open-flow nitrogen cryostat (Cosier & Glazer, 1986) operating at 100.0 (1) K.
Geometry. All e.s.d.'s (except the e.s.d. in the dihedral angle between two l.s. planes) are estimated using the full covariance matrix. The cell e.s.d.'s are taken into account individually in the estimation of e.s.d.'s in distances, angles and torsion angles; correlations between e.s.d.'s in cell parameters are only used when they are defined by crystal symmetry. An approximate (isotropic) treatment of cell e.s.d.'s is used for estimating e.s.d.'s involving l.s. planes.
Refinement. Refinement of *F*^2^ against ALL reflections. The weighted *R*-factor *wR* and goodness of fit *S* are based on *F*^2^, conventional *R*-factors *R* are based on *F*, with *F* set to zero for negative *F*^2^. The threshold expression of *F*^2^ > σ(*F*^2^) is used only for calculating *R*-factors(gt) *etc*. and is not relevant to the choice of reflections for refinement. *R*-factors based on *F*^2^ are statistically about twice as large as those based on *F*, and *R*- factors based on ALL data will be even larger.

### Fractional atomic coordinates and isotropic or equivalent isotropic displacement parameters (Å^2^)

**Table:**

	*x*	*y*	*z*	*U*_iso_*/*U*_eq_	
O1	0.28632 (10)	−0.12818 (8)	0.76247 (7)	0.01976 (19)	
O2	0.46453 (10)	0.00161 (9)	0.76921 (7)	0.0212 (2)	
O3	0.66947 (10)	0.34436 (10)	1.03221 (8)	0.0273 (2)	
O4	0.55970 (10)	0.42674 (9)	1.10945 (8)	0.0228 (2)	
N1	0.33488 (11)	0.29963 (10)	1.09858 (8)	0.0181 (2)	
N2	0.56820 (11)	0.34349 (10)	1.05006 (8)	0.0184 (2)	
C1	0.46344 (13)	0.16060 (11)	0.92632 (9)	0.0165 (2)	
H1A	0.5353	0.1764	0.9095	0.020*	
C2	0.45671 (12)	0.24143 (11)	1.00074 (9)	0.0154 (2)	
C3	0.34771 (13)	0.22195 (11)	1.02854 (9)	0.0153 (2)	
C4	0.24935 (13)	0.11392 (12)	0.97700 (9)	0.0169 (2)	
H4A	0.1767	0.0968	0.9929	0.020*	
C5	0.25813 (13)	0.03416 (12)	0.90452 (10)	0.0173 (2)	
H5A	0.1924	−0.0362	0.8730	0.021*	
C6	0.36523 (13)	0.05748 (11)	0.87723 (9)	0.0161 (2)	
C7	0.37913 (13)	−0.02319 (12)	0.79882 (10)	0.0173 (2)	
C8	0.29118 (15)	−0.20792 (13)	0.68146 (11)	0.0239 (3)	
H8A	0.3826	−0.2563	0.7107	0.029*	
H8B	0.2825	−0.1497	0.6259	0.029*	
C9	0.16782 (18)	−0.30447 (14)	0.63980 (12)	0.0324 (3)	
H9A	0.1677	−0.3582	0.5858	0.049*	
H9B	0.0780	−0.2555	0.6114	0.049*	
H9C	0.1780	−0.3620	0.6953	0.049*	
C10	0.23046 (13)	0.27774 (12)	1.13077 (10)	0.0175 (2)	
C11	0.11792 (14)	0.37011 (13)	1.10012 (11)	0.0227 (3)	
H11A	0.1090	0.4418	1.0567	0.027*	
C12	0.01887 (15)	0.35537 (14)	1.13428 (12)	0.0281 (3)	
H12A	−0.0559	0.4176	1.1143	0.034*	
C13	0.03126 (15)	0.24804 (14)	1.19810 (12)	0.0287 (3)	
H13A	−0.0359	0.2374	1.2202	0.034*	
C14	0.14412 (16)	0.15626 (14)	1.22907 (12)	0.0269 (3)	
H14A	0.1528	0.0846	1.2724	0.032*	
C15	0.24447 (15)	0.17090 (12)	1.19555 (11)	0.0218 (3)	
H15A	0.3202	0.1095	1.2165	0.026*	
H1N1	0.3899 (19)	0.3693 (16)	1.1227 (13)	0.030 (4)*	

### Atomic displacement parameters (Å^2^)

**Table:**

	*U*^11^	*U*^22^	*U*^33^	*U*^12^	*U*^13^	*U*^23^
O1	0.0247 (5)	0.0208 (4)	0.0188 (5)	0.0008 (3)	0.0147 (4)	−0.0030 (3)
O2	0.0237 (5)	0.0249 (4)	0.0222 (5)	0.0049 (3)	0.0169 (4)	0.0034 (4)
O3	0.0211 (5)	0.0350 (5)	0.0342 (6)	−0.0065 (4)	0.0202 (4)	−0.0053 (4)
O4	0.0259 (5)	0.0229 (4)	0.0250 (5)	−0.0052 (4)	0.0169 (4)	−0.0054 (4)
N1	0.0189 (5)	0.0194 (5)	0.0218 (6)	−0.0037 (4)	0.0146 (4)	−0.0049 (4)
N2	0.0176 (5)	0.0211 (5)	0.0193 (5)	−0.0010 (4)	0.0113 (4)	0.0013 (4)
C1	0.0172 (5)	0.0188 (5)	0.0176 (6)	0.0039 (4)	0.0119 (5)	0.0048 (4)
C2	0.0151 (5)	0.0172 (5)	0.0163 (6)	0.0000 (4)	0.0096 (5)	0.0011 (4)
C3	0.0153 (5)	0.0176 (5)	0.0153 (6)	0.0018 (4)	0.0092 (4)	0.0011 (4)
C4	0.0157 (5)	0.0211 (5)	0.0172 (6)	−0.0009 (4)	0.0107 (5)	−0.0005 (4)
C5	0.0176 (5)	0.0191 (5)	0.0167 (6)	−0.0001 (4)	0.0098 (5)	−0.0005 (4)
C6	0.0182 (5)	0.0187 (5)	0.0146 (6)	0.0036 (4)	0.0107 (5)	0.0025 (4)
C7	0.0195 (6)	0.0183 (5)	0.0162 (6)	0.0050 (4)	0.0106 (5)	0.0037 (4)
C8	0.0314 (7)	0.0249 (6)	0.0208 (7)	0.0044 (5)	0.0171 (6)	−0.0038 (5)
C9	0.0428 (9)	0.0279 (7)	0.0307 (8)	−0.0050 (6)	0.0217 (7)	−0.0078 (6)
C10	0.0157 (5)	0.0222 (5)	0.0183 (6)	−0.0038 (4)	0.0113 (5)	−0.0063 (5)
C11	0.0181 (6)	0.0273 (6)	0.0227 (7)	0.0004 (5)	0.0104 (5)	−0.0031 (5)
C12	0.0181 (6)	0.0356 (7)	0.0334 (8)	−0.0002 (5)	0.0151 (6)	−0.0101 (6)
C13	0.0256 (7)	0.0362 (7)	0.0356 (8)	−0.0118 (6)	0.0239 (6)	−0.0167 (6)
C14	0.0348 (8)	0.0266 (6)	0.0314 (8)	−0.0086 (5)	0.0256 (7)	−0.0073 (6)
C15	0.0245 (6)	0.0217 (6)	0.0259 (7)	−0.0012 (5)	0.0176 (6)	−0.0033 (5)

### Geometric parameters (Å, º)

**Table:**

O1—C7	1.3470 (15)	C6—C7	1.4764 (16)
O1—C8	1.4582 (14)	C8—C9	1.485 (2)
O2—C7	1.2165 (13)	C8—H8A	0.9700
O3—N2	1.2325 (12)	C8—H8B	0.9700
O4—N2	1.2415 (13)	C9—H9A	0.9600
N1—C3	1.3518 (14)	C9—H9B	0.9600
N1—C10	1.4293 (14)	C9—H9C	0.9600
N1—H1N1	0.858 (17)	C10—C15	1.3852 (17)
N2—C2	1.4468 (15)	C10—C11	1.3904 (17)
C1—C6	1.3789 (17)	C11—C12	1.3875 (17)
C1—C2	1.3923 (15)	C11—H11A	0.9300
C1—H1A	0.9300	C12—C13	1.384 (2)
C2—C3	1.4262 (15)	C12—H12A	0.9300
C3—C4	1.4218 (16)	C13—C14	1.388 (2)
C4—C5	1.3727 (16)	C13—H13A	0.9300
C4—H4A	0.9300	C14—C15	1.3935 (17)
C5—C6	1.4071 (15)	C14—H14A	0.9300
C5—H5A	0.9300	C15—H15A	0.9300
			
C7—O1—C8	115.13 (9)	O1—C8—H8A	110.2
C3—N1—C10	124.35 (10)	C9—C8—H8A	110.2
C3—N1—H1N1	117.8 (11)	O1—C8—H8B	110.2
C10—N1—H1N1	117.8 (11)	C9—C8—H8B	110.2
O3—N2—O4	122.02 (11)	H8A—C8—H8B	108.5
O3—N2—C2	118.75 (10)	C8—C9—H9A	109.5
O4—N2—C2	119.23 (9)	C8—C9—H9B	109.5
C6—C1—C2	121.06 (10)	H9A—C9—H9B	109.5
C6—C1—H1A	119.5	C8—C9—H9C	109.5
C2—C1—H1A	119.5	H9A—C9—H9C	109.5
C1—C2—C3	121.55 (11)	H9B—C9—H9C	109.5
C1—C2—N2	116.46 (10)	C15—C10—C11	120.29 (11)
C3—C2—N2	121.97 (10)	C15—C10—N1	121.04 (11)
N1—C3—C4	120.59 (10)	C11—C10—N1	118.58 (11)
N1—C3—C2	123.68 (11)	C12—C11—C10	120.00 (13)
C4—C3—C2	115.72 (10)	C12—C11—H11A	120.0
C5—C4—C3	122.11 (10)	C10—C11—H11A	120.0
C5—C4—H4A	118.9	C13—C12—C11	120.03 (13)
C3—C4—H4A	118.9	C13—C12—H12A	120.0
C4—C5—C6	120.86 (11)	C11—C12—H12A	120.0
C4—C5—H5A	119.6	C12—C13—C14	119.90 (11)
C6—C5—H5A	119.6	C12—C13—H13A	120.0
C1—C6—C5	118.69 (10)	C14—C13—H13A	120.0
C1—C6—C7	117.78 (10)	C13—C14—C15	120.36 (13)
C5—C6—C7	123.53 (11)	C13—C14—H14A	119.8
O2—C7—O1	122.94 (11)	C15—C14—H14A	119.8
O2—C7—C6	124.18 (11)	C10—C15—C14	119.41 (12)
O1—C7—C6	112.88 (9)	C10—C15—H15A	120.3
O1—C8—C9	107.49 (10)	C14—C15—H15A	120.3
			
C6—C1—C2—C3	−0.58 (18)	C4—C5—C6—C7	−179.04 (11)
C6—C1—C2—N2	177.85 (11)	C8—O1—C7—O2	−2.29 (17)
O3—N2—C2—C1	−7.34 (16)	C8—O1—C7—C6	177.49 (10)
O4—N2—C2—C1	173.15 (11)	C1—C6—C7—O2	−5.67 (18)
O3—N2—C2—C3	171.07 (11)	C5—C6—C7—O2	174.66 (12)
O4—N2—C2—C3	−8.44 (17)	C1—C6—C7—O1	174.56 (10)
C10—N1—C3—C4	3.33 (18)	C5—C6—C7—O1	−5.11 (17)
C10—N1—C3—C2	−177.07 (11)	C7—O1—C8—C9	−171.43 (11)
C1—C2—C3—N1	−178.51 (11)	C3—N1—C10—C15	72.36 (17)
N2—C2—C3—N1	3.15 (18)	C3—N1—C10—C11	−110.92 (14)
C1—C2—C3—C4	1.12 (17)	C15—C10—C11—C12	−0.1 (2)
N2—C2—C3—C4	−177.22 (10)	N1—C10—C11—C12	−176.86 (12)
N1—C3—C4—C5	179.17 (12)	C10—C11—C12—C13	−0.6 (2)
C2—C3—C4—C5	−0.47 (17)	C11—C12—C13—C14	0.9 (2)
C3—C4—C5—C6	−0.72 (19)	C12—C13—C14—C15	−0.5 (2)
C2—C1—C6—C5	−0.65 (18)	C11—C10—C15—C14	0.49 (19)
C2—C1—C6—C7	179.67 (11)	N1—C10—C15—C14	177.15 (12)
C4—C5—C6—C1	1.29 (18)	C13—C14—C15—C10	−0.2 (2)

### Hydrogen-bond geometry (Å, º)

**Table:**

*D*—H···*A*	*D*—H	H···*A*	*D*···*A*	*D*—H···*A*
N1—H1*N*1···O4	0.859 (18)	2.00 (2)	2.6375 (17)	130.6 (17)
N1—H1*N*1···O2^i^	0.858 (17)	2.288 (16)	2.9411 (14)	133.0 (14)
C15—H15*A*···O2^ii^	0.93	2.45	3.342 (2)	160

Symmetry codes: (i) *x*, −*y*+1/2, *z*+1/2; (ii) −*x*+1, −*y*, −*z*+2.
